# Potential therapeutic targets of *TP53* gene in the context of its classically canonical functions and its latest non-canonical functions in human cancer

**DOI:** 10.18632/oncotarget.24611

**Published:** 2018-03-23

**Authors:** Toshimichi Tanaka, Masahiko Watanabe, Keishi Yamashita

**Affiliations:** ^1^ Department of Surgery, Kitasato University School of Medicine, Minami-ku, Sagamihara, Kanagawa, 252-0374, Japan; ^2^ Division of Advanced Surgical Oncology, Department of Research and Development Center for New Medical Frontiers, Minami-ku, Sagamihara, Kanagawa, 252-0374, Japan

**Keywords:** p53, mutation, gain of function, non-canonical function, carcinogenesis

## Abstract

In normal tissue, p53 protein has a wide range of functions involving cell homeostasis; its mutation, however, permits a carcinogenic acquisition of function. *TP53* gene mutation is a major genomic aberration in various human cancers and is a critical event in the multi-step carcinogenesis process. *TP53* mutation is clinically relevant for the molecular classification of carcinogenesis, as most recently described rigorously by the Cancer Genome Atlas Research Network. *TP53* gene mutation has been considered to work as a tumor suppressor gene through the loss of its transcriptional activity, which is designated as a canonical function. However, in cancer patients with mutant *TP53*, mutated p53 protein is frequently overexpressed, suggesting the activation of an oncogenic process through a gain of function (GOF). As part of this GOF, molecular mechanisms explaining the non-canonical function of *TP53* gene abnormality have been reported, in which mutant p53 unconventionally binds with various critical molecules suppressing oncogenic properties, such as p63 and p73. Moreover, mutant *TP53* gene-targeted therapy has been rigorously developed, and promising clinical trials have been started. In this study, we summarize the novel aspects of mutant p53 and describe its prominent therapeutic potentials in human cancer.

## INTRODUCTION

The *TP53* gene has 11 exons and produces an mRNA of 2.2-2.5 kb. The wild-type (WT) p53 protein has a wide range of functions involved in cell homeostasis, including the cell cycle, DNA maintenance, and apoptosis, while mutant p53 protein is seen in most cancers. Therefore, the *TP53* gene has been referred to as the “Guardian of the Genome” [1] or the “Death Star” [2]. In 1981, it was discovered that the p53 protein, which is a 53,000-dalton protein, accumulates along with proto-virus protein [3]. The *TP53* gene was initially thought to be an oncogene, since mutant p53 protein was overexpressed in cancer cells. However, it was subsequently confirmed that the *TP53* gene is located on the short arm of chromosome 17, which is frequently deleted in human cancer [4, 5]. In addition, mutation of the *TP53* gene was confirmed to cause Li-Fraumeni syndrome, which leads to the development of various primary cancers at a young age [6]. Thus, research on the function of p53 protein tended to focus on its role as a tumor suppressor gene.

The canonical functions of the *TP53* gene are based on its transcriptional activity [7] and its promotion of the transactivation of the *p21^WAF1^* gene, which is involved in cell cycle arrest [8]. After crystal structure analyses became possible, the DNA binding region of p53 protein was found to be the location of many point mutations associated with tumorigenesis. Therefore, mutation of the DNA binding region of p53 protein was considered to lead to a dysfunction in the transactivation of normal p53 protein [9]. Distinctive functional abnormalities occur depending on the type and locations of *TP53* gene mutation. *TP53* gene alterations often consist of missense mutations, but infrequent nonsense or frameshift mutations do occur. Missense mutations are usually observed in exons 5-8, while nonsense and frameshift mutations are more frequent in exons 4, 9, and 10 [10]. Single point mutations are located in the *TP53* DNA-binding domain for more than 95% of all reported carcinogenic mutations [11]. Mutations at these residues can be categorized as contact (R273H, R248Q and R248W) or structural (R175H, G245S, R249S and R282H) mutants, depending on whether the residues have a role in direct DNA contact or in the maintenance of the p53 structure. Reportedly, contact mutant proteins are destabilizing and often exhibit a nuclear distribution in response to genotoxic stress, while structural mutant proteins are typically distributed around the nucleus of protein aggregates [12].

**Figure 1:**
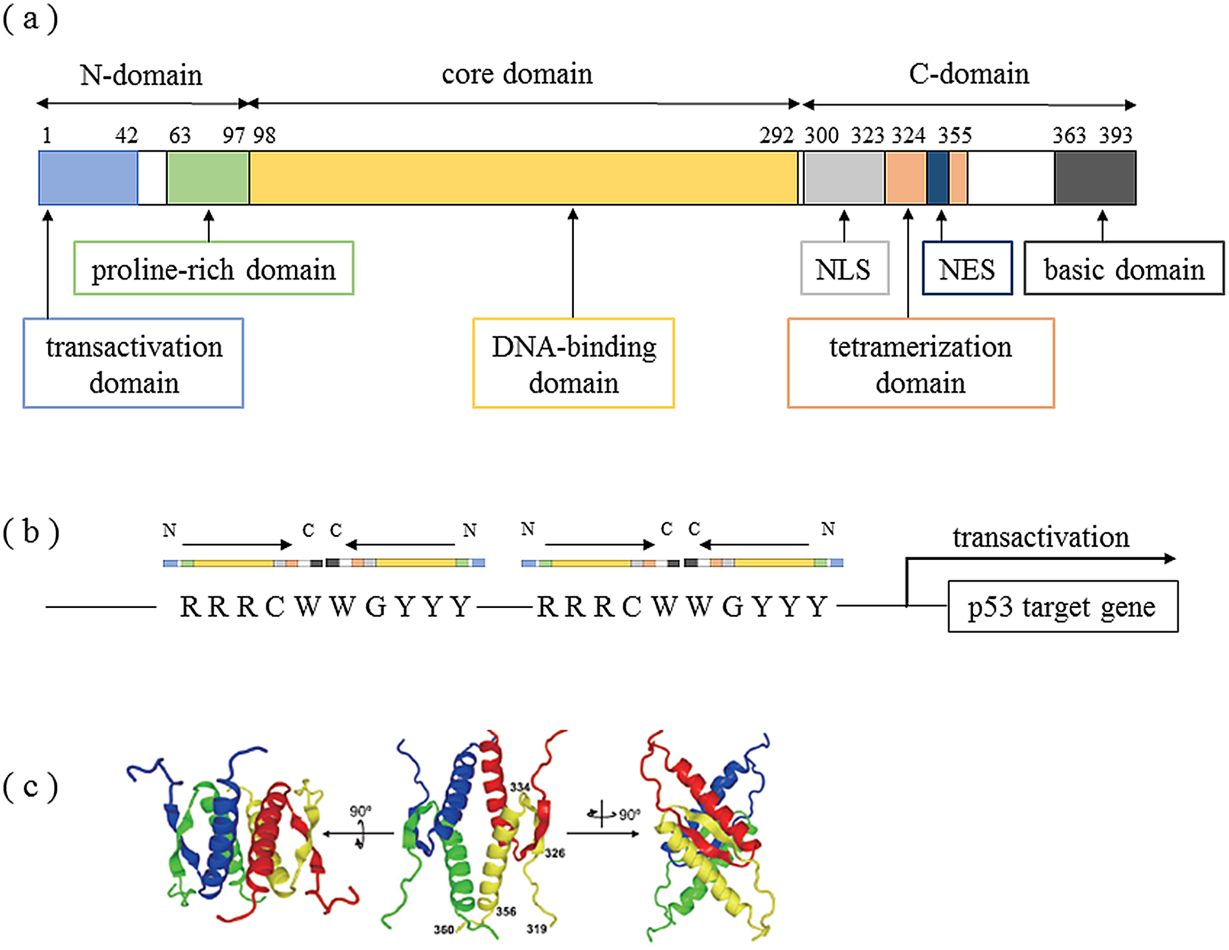
Structure of the tetramer formation domain in p53 protein **(a)** Domain structure of p53. Human p53 is composed of 393 amino acid residues and has a transactivation domain, proline-rich domain, DNA-binding domain, nuclear localization signal area, tetramerization domain, nuclear export signal area, and basic domain. Modified from Ref. 10. **(b)** Transcriptional activation mechanisms of target genes by p53 protein. Various stresses activate p53, which then recognizes the sequence RRRCWWGYYY (R: purine base, Y: pyrimidine base, W: adenine or thymine) in the nucleus and combines with the sequence to form a tetramer. **(c)** NMR (nuclear magnetic resonance) structure of tetramerization domains. The tetramerization domain contains a [β strand-turn-α helix] structure and forms a tetramer from 2 antiparallel b sheets and 4 helix bundles. Citation from Ref. 15.

A detailed sequence analysis revealed the domains that are involved in protein modifications of p53 protein, including a transactivation domain, an apoptosis-related domain within a proline-rich domain, a tetramerization-related domain, and a basic domain [13]. Moreover, the C-terminus contains a nuclear localization signal (NLS) and a nuclear export signal (NES), which are involved in the regulation of the appropriate subcellular localization of p53 function (Figure 1a) [14]. WT p53 protein forms a tetramer and performs transcriptional control by binding to specific sequence sites of genomic DNA (Figure 1b, 1c) [15]. Cross-linking of various post-transcriptional modifications (PTMs) have been reported to affect both the stability and activation of p53, including tetramer formation and localization [16–18], and these PTMs can be functionally divided into two groups: ones that stabilize and activate p53 (phosphorylation, acetylation, and methylation of K372), and ones that, in contrast, lead to the degradation and inactivation of p53 (ubiquitination, neddylation, sumoylation, and methylation of certain lysines). Although the functional analysis of PTMs has been much discussed, the significance of many PTMs, including ubiquitination and acetylation, has largely remained unclear.

In this review article, we would like to provide the rationale for a review on mutant p53 from a clinical point of view, including its clinical relevance and potential therapeutic targets of the *TP53* gene in the context of its classically canonical functions and its latest non-canonical functions in human cancer.

### Canonical functions of WT p53 protein and its transcriptional target genes

Although normal cells retain only a small amount of p53 protein, levels are increased during times of stress [19]. Under basal conditions, the dysfunctionality of p53 protein occurs mainly by binding to MDM2 and MDM4 [20, 21]. Heterodimers of MDM2 and MDM4 bind to p53 through their N-terminus and activate the E3 ligase activity of MDM2 to induce proteasomal degradation of the p53 protein [22–24]. MDM4 inhibits both the degradation and transactivity of p53, thereby becoming a target of the stress signal to disengage the p53/MDM2 feedback loop for appropriate p53 responses to stresses [23, 25–27]. The balance of p53 activation-related genes can have a very important role in the mechanism of carcinogenesis, since *MDM2* gene amplification has been recognized in tumors like sarcoma, in which *TP53* alterations are less frequent [28].

The major canonical functions of WT p53 involve growth arrest, apoptosis, and DNA repair. Cell cycle arrest by WT p53 is mediated through transcriptional target genes such as *p21*, *14-3-3σ* and *reprimo*. p21 suppresses both the G1/S and the G2/M phases [8, 29], while 14-3-3σ and reprimo suppress the G2/M phase [30, 31]. During apoptosis, the *Bax* gene is directly induced by the p53 protein. [32, 33]. Bax promotes the release of cytochrome c in mitochondria and causes apoptosis through the activation of multiple caspases. Moreover, p53 can directly induce the expression of the *PUMA* gene, the protein of which can bind to Bcl-2 or Bcl-xL through the Bcl-2 homology 3 (BH3) domain to induce Bax [34]. *Noxa* also encodes a BH3 domain and causes apoptosis through the same mechanism [35]. In addition, p53 can induce the *KILLER/DR5* gene, with the resulting DR5 protein activating Bax through FADD [36]. Apoptosis is strictly controlled by the numerous overlapping layers of p53 functions. DNA repair genes are also strictly increased in the p53 canonical pathway through the induction of the expressions of the *GADD45*, *XPC*, and *p53 R2* genes [37–39]. When the cells suffer minor DNA damage, the aberrations are repaired during cell cycle arrest, enabling the cells to survive while destroying cancer cells that have received major DNA damage.

**Figure 2:**
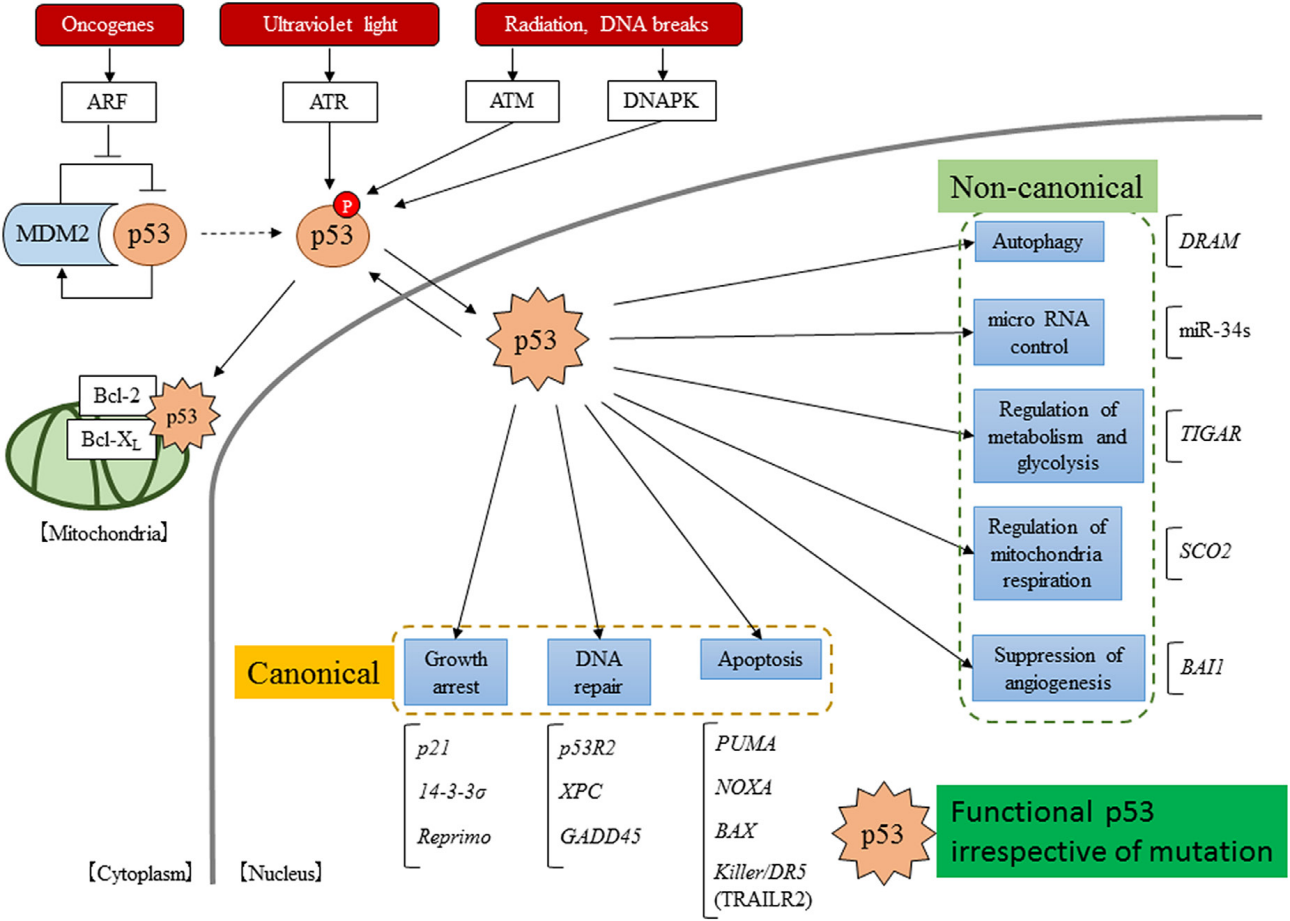
Scheme of the p53 pathway p53 is activated by a variety of stressors and p53 suppressor genes, such as MDM2. In the nucleus, activated p53 induces the transcriptional activation of many target genes. Moreover, in the cytoplasm, activated p53 has been reported to localize directly in mitochondria and to induce non-transferable dependent apoptosis.

On the other hand, several recent reports have indicated that WT and mutant p53 protein have many non-canonical functions, the detailed mechanisms of which are not all clear. The targets of the WT p53 protein that are involved in non-canonical functions are the *BAI1* gene (brain-specific angiogenesis inhibitor 1) [40, 41], which is associated with the inhibition of angiogenesis; the *TIGAR* gene (TP53-induced glycolysis and apoptosis regulator), which is associated with the inhibition of glycolysis and protection against oxidative stress [42]; the *SCO2* gene (synthesis of cytochrome C oxidase 2), which is associated with mitochondrial respiration [43, 44]; the *DRAM* gene (damage-regulated autophagy modulator), which is associated with the positive regulation of autophagy [45]; and miR-34s associated with the production of cell-cycle arrest and an increase in apoptosis [46]. A summary of the molecular mechanisms of p53 functions is shown in Figure 2.

### “Gain of function” associated with *TP53* mutation explains a novel p53 non-canonical function

Mutant p53 protein is frequently overexpressed in cancer cells [47], and thus the mutation of the *TP53* gene results in not only a loss of function of WT p53, but also a gain of function (GOF). Overexpression of the mutant p53 protein has been presumed to be related to the new acquisition of oncogenic functions.

The R175H and R273H mutations, which are mutation hot spots for the *TP53* gene, were confirmed to accelerate metastasis via a GOF in various mouse models. Liu et al. reported that mice with the R175H mutation exhibit increased tumor formation and metastasis and a simultaneous loss of WT p53 functions [48]. Lang et al. also reported that embryonic fibroblasts from p53R175H mutant mice showed enhanced cell proliferation, DNA synthesis, and a metastatic status [49]. Similarly, mice with the R273H mutation exhibited tumor growth [50], and Heinlein et al. revealed that genomic instability was not accompanied by such tumorigenic changes [51]. It has become obvious that *TP53* mutation elicits GOF effects.

**Figure 3:**
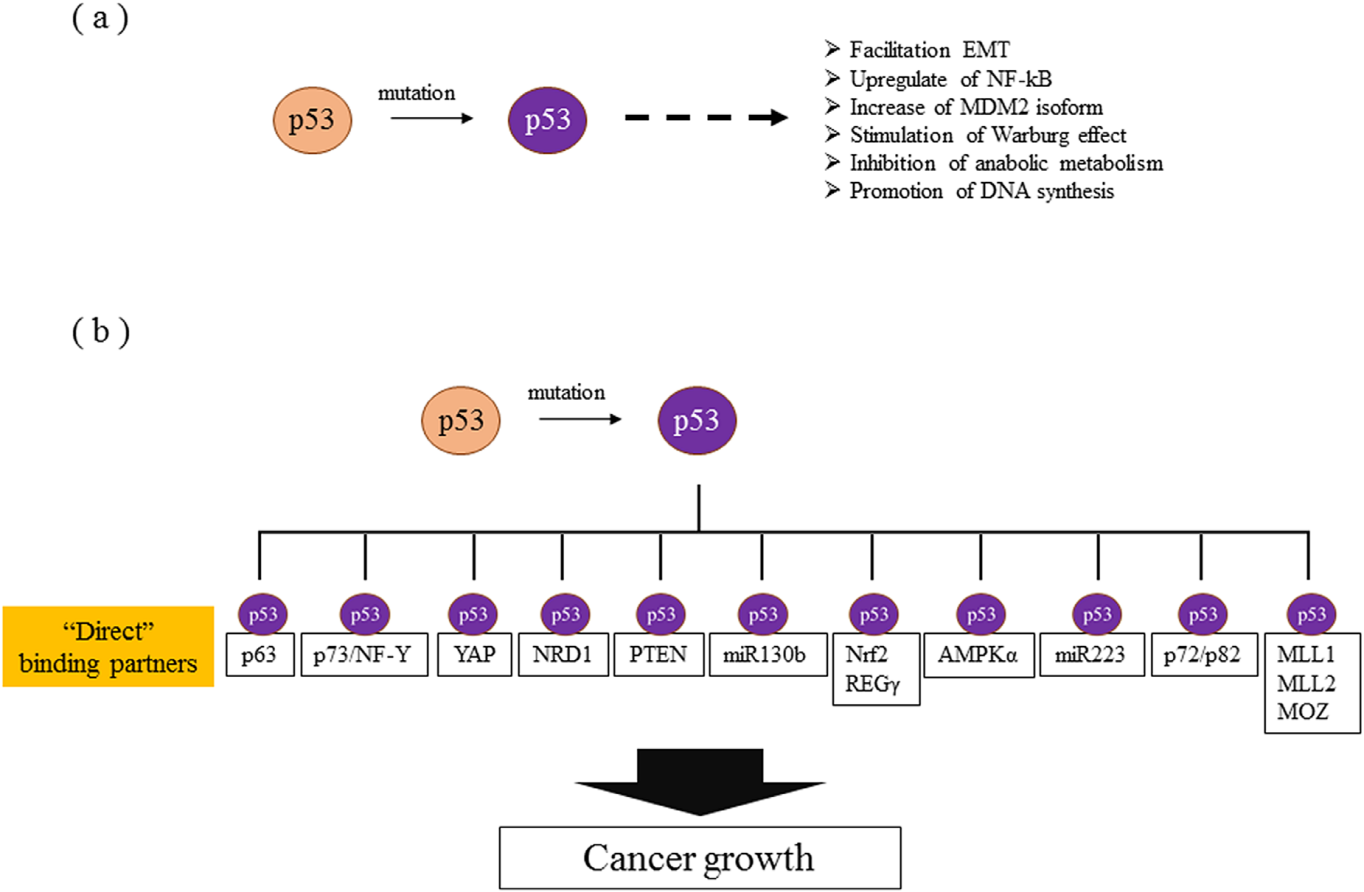
Molecular mechanisms of gain of function **(a)** Non-canonical functions whose direct interactions with mutant p53 have not been elucidated. **(b)** Mutant p53s promote the transcriptional activation of various genes. The molecules reported to bind directly to mutant p53 are listed.

Mutant p53 has been demonstrated to facilitate non-canonical GOF effects, such as the epithelial mesenchymal transition (EMT) [52, 53], the activation of NF-kβ [54], increases in chemo-resistance [55] and radio-resistance [56], assistance in the resolution of the proteasome [57], an increase in the MDM2 isoform [58], the stimulation of aerobic glycolysis [59], the inhibition of anabolic metabolism [60], the promotion of DNA synthesis [61, 62], histone modification [63], and the enhancement of integrin and Rho GTPase signaling [64]. Therefore, some of these non-canonical functions can be explained, at least in part, by a GOF of mutant p53 (Figure 3a).

The precise molecular mechanisms of mutant p53 have been lately studied more deeply. Mutant p53, but not WT p53, can directly bind to isoforms of p63, which is a p53 family protein [65, 66]. Muller et al. reported that the direct inhibition of p63 by mutant p53 is involved in cell proliferation and invasion via the enhanced expression of α5β1-integrin and EGFR signals in tumor cells [67]. To identify the mechanism involved in such non-canonical functions accurately, we need to determine the direct binding partner, such as p63, with mutant p53 protein to explain the GOF. p73, a p53 family member other than p63, is also a direct binding partner of mutant p53 [68]. Although p63 and p73 are not directly associated with tumorigenesis in normal cells, in the presence of mutant p53, they can become a transcription target and are related to the progression of tumors. Indeed, there is a report that mutant p53 induces PDGFRb (platelet derived growth factor receptor beta) through a cell-autonomous mechanism involving the inhibition of a p73/NF-Y complex that represses PDGFRb expression in p53-deficient, noninvasive cells [69].

Direct potential candidate partners with mutant p53 have been recently and rigorously reported, as shown in Figure 3b. PTEN enhances mutant p53 protein levels via the inhibition of mutant p53 degradation by Mdm2, and this is considered to be mediated through direct protein binding between mutant p53 and PTEN [70]. The Rab11 effector protein, a Rab-coupling protein, was also reported to be mediated through a mechanism independent of p63 and to result in the enhancement of α5β1-integrin and EGFR in tumor cells [67]. Coffill et al. proposed a different mechanism for mutant p53-driven invasion: the interaction of p53 R273H with nardilysin (NRD1) promotes an invasive response to heparin binding-epidermal growth factor-like growth factor that is p53R273H-dependant but that does not require Rab coupling protein or p63 [71]. Mutant p53 also reportedly exerts oncogenic functions and promotes EMT in endometrial cancer by binding directly to the promoter of miR-130b, a negative regulator of ZEB1, and inhibiting its transcription [53]. Moreover, mutant p53 protein prevents Smad3/N-CoR complex formation on the REGγ promoter, which enhances the activity of the REGγ-20S proteasome pathway and contributes to mutant p53 GOF [57]. In addition, mutant p53 protein binds the miR-223 promoter and reduces its transcriptional activity, resulting in chemo-resistance via the upregulation of STMN-1 [55]. Furthermore, some reports have indicated that mutant 53 protein, but not WT p53 protein, preferentially binds to the AMPKα subunit and inhibits AMPK activation under conditions of energy stress [60].

p53 mutants also bind to and upregulate chromatin regulatory genes, including the methyltransferases MLL1 (also known as KMT2A), MLL2 (also known as KMT2D), and the acetyltransferase MOZ (also known as KAT6A or MYST3), resulting in genome-wide increases in histone methylation and acetylation [63]. Mutant p53 also binds and sequesters RNA helicases p72/82 from the microprocessor complex, interfering with the Drosha-pri-miRNAs association [72]. Recently, mutant p53 was reported to cooperate with Nrf2 (NFE2L2) to activate proteasome gene transcription, resulting in resistance to the proteasome inhibitor carfilzomib in cancer cells [73]. In addition, the p53 mutant protein physically interacts with Yes-associated protein (YAP), a key transcriptional regulator controlling organ growth, tissue homeostasis, and cancer [74].

### Clinical relevance of the *TP53* gene mutation during molecular carcinogenesis

Vogelstein et al. proposed that genetic aberrations accumulate in accordance with the precancerous-cancer sequence [75]. Genetic aberration occurs in a multi-step carcinogenesis process that includes genomic mutations as well as other somatic changes such as chromosomal instability (CIN), microsatellite instability (MSI), and a CpG island methylation phenotype (CIMP) [76]. The mutation of the *TP53* gene is induced by activation-induced cytidine deaminase (AID) through chronic inflammation in gastric cancer (GC), liver cancer, and colorectal cancer (CRC) [77–82]. Thus, a common molecular mechanism explaining *TP53* gene mutagenesis in the context of chronic inflammation is assumed to exist.

Molecular multi-step carcinogenesis has been commonly recognized in CRC. The mutation of the *TP53* gene is thought to occur during the latter half of carcinogenesis (highly atypical polyp) and to cause malignant transformation in CRC [75]. Actually, the TP53 mutation was found in 58% of CRC and was accompanied by a high frequency of APC gene mutation (82%) and KRAS gene mutation (46%) [83]. The CIN phenotype includes chromosomal amplification or deletion and has been observed in about 80% of CRCs [84]. The amplification of chromosome 8q, 13q, or 20q and the deletion of chromosome 8p, 15q, 17p, or 18q are correlated with carcinogenesis in high-grade adenoma [85]. Although the *TP53* gene mutation coexists with CIN in many cases of CRC and was thought to elicit CIN, experiments with knockout WT *TP53* show no change in CIN [86]. Therefore, the loss of p53 function has recently been considered to result from CIN. On the other hand, an inverse correlation between the frequency of the occurrence of CIN and MSI/CIMP has been reported [87]. In other words, the mutation of the *TP53* gene is rarely detected in lesions with CIMP, while the mutation of the *TP53* gene and CIN are common changes associated with carcinogenesis in CIMP-negative tumors.

**Figure 4:**
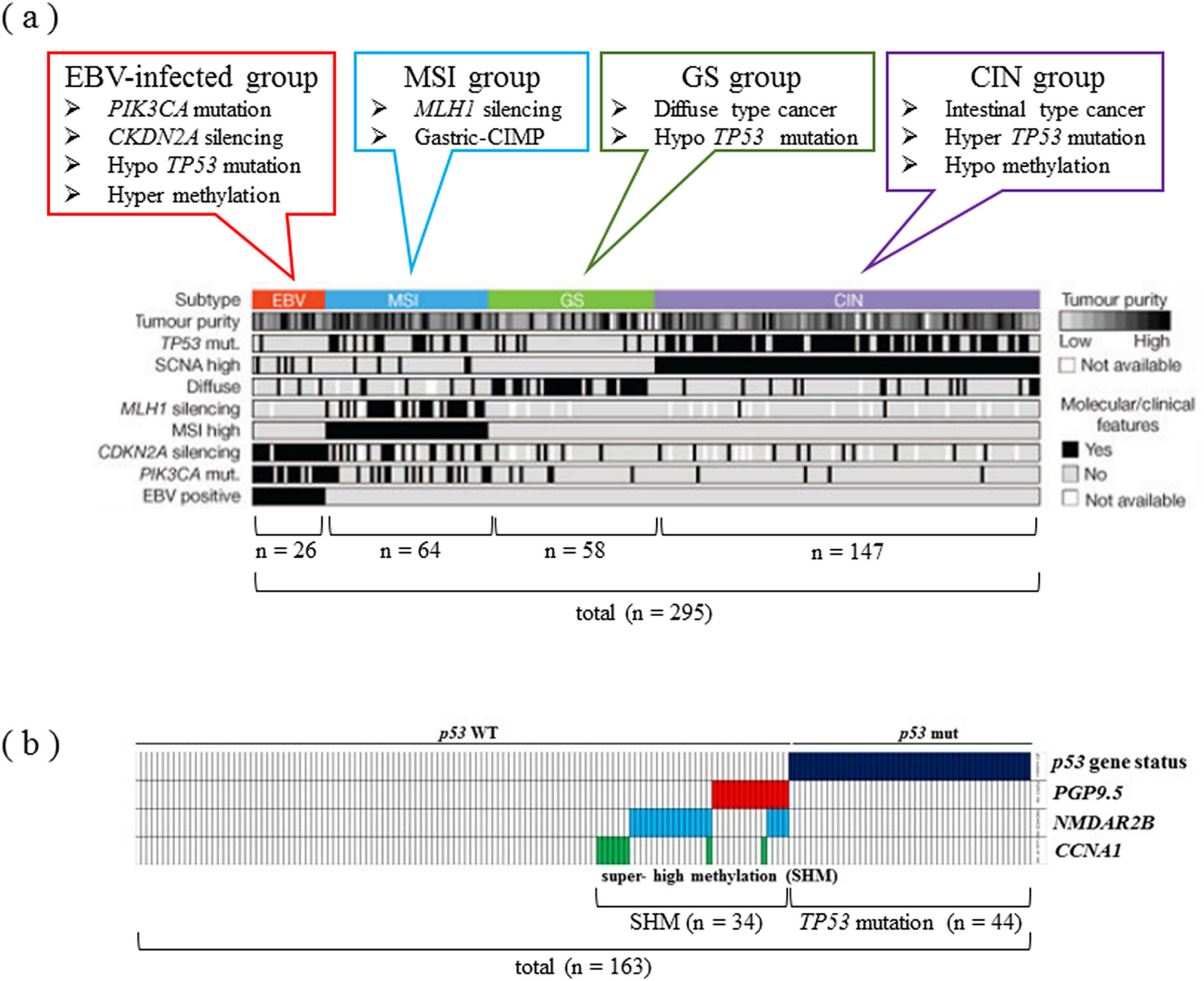
Classification and features of carcinogenic mechanisms through a comprehensive molecular search in GC **(a)** Comprehensive molecular search of 295 gastric adenocarcinoma cases. In the EBV-infected group, *PI3K* mutation and *p16* silencing are characteristic genomic features, while the MSI group has low-level gene expressions of *MLH1*. *TP53* gene mutations are concentrated in the CIN group, which are often intestinal-type cancer. Modified from Ref. 88. **(b)** Epigenetic control of cancer suppressor genes related to the *TP53* pathway in GC. The super high methylation of *PGP9.5*, *NMDAR2B*, and *CCNA1* genes is only seen in GC patients with WT *TP53*. The *TP53* pathway-aberration group, including mutant *TP53* and SHM of the 3 genes, is frequently observed in intestinal-type GC. Modified from Ref. 87.

This inverse correlation has also been observed in GC, especially intestinal-type cancer [88]. In GC, diffuse type and intestinal type cancers are mixed, and the latter is often triggered by chronic inflammation associated with *Helicobacter pylori* infection. For this reason, GC has a lower frequency of *TP53* gene mutation than CRC [88]. Recently, the comprehensive molecular characteristics of GC have been clarified, a GC can now be classified into four types according to its molecular features (Figure 4a) [89]. These subgroups consist of CIN, genomic stable (GS), MSI, and EBV infection, and *TP53* gene mutations are concentrated in the CIN group, which are often intestinal-type cancers. Intestinal-type GC has a similar oncogenic process to CRC and can be triggered by chronic inflammation. In contrast, diffuse-type tumor, the major phenotype of GC, has been reported to occur because of E-cadherin mutation, with only a few tumors carrying *TP53* gene mutations.

Chronic inflammation induced by *H. pylori* infection has been demonstrated to be related to the DNA methylation of tumor suppressor genes in GC [90]. Moreover, it has been suggested that epigenetic control is associated with the malignant conversion of tumors, and the “super high methylation (SHM)” of 3 tumor suppressor genes (*PGP9.5*, *NMDAR2B*, and *CCNA1*), which are related to the *TP53* pathways (Figure 4b), was identified [88]. The SHM of these genes was only recognized in GC patients with WT *TP53*, which is reminiscent of the inverse correlation between *TP53* mutation/CIN and MSI/CIMP in GC, similar to CRC. In this study, the p53 pathway-aberration group, including *TP53* mutation and the SHM of the 3 genes, was frequently observed in intestinal-type GC.

Breast cancer is known to have different *TP53* mutation rates according to the basic therapeutic subtypes, such as the luminal A, luminal B, HER2-enriched (HER2E) and triple-negative (TN) subtypes [91]. *TP53* mutations in TN tumors were the most frequent among the subtypes (80%), while a few mutations of other cancer-related genes including *PIK3CA* were also present. The luminal A subtype had fewer *TP53* mutations (12%), compared with the TN subtype, but had numerous *PIK3CA* mutations (45%). The luminal B subtype had mutations of *TP53* and *PIK3CA* (29% each), whereas the HER2E subtype had a hybrid pattern with a high frequency of *TP53* (72%) and *PIK3CA* (39%) mutations.

The genome profiles of TN breast cancer and high-grade serous ovarian cancer (HGS-OvCa) were recently reported [91]. HGS-OvCa has a high TP53 mutation rate (96%), but a few somatic mutations of BRCA1/2 are present in an additional 3% of cases [92]. On the other hand, clear-cell OvCa has a low rate of *TP53* mutations but exhibits recurrent *PIK3CA* and *ARID1A* mutations [93–95]. Endometrioid OvCa has a lower rate of *TP53* and more frequent *CTTNB1*, *ARID1A*, and *PIK3CA* mutations, while *KRAS* mutations are prevalent in mucinous OvCa [94–96].

Similar to TN breast cancer and HGS-OvCa, almost all lung squamous cell carcinoma (lung SqCC) exhibited frequent somatic mutations of *TP53* (81%), while both *EGFR* and *KRAS* mutations were almost absent [97]. Lung SqCC also had frequent alterations in the *CDKN2A*/*RB1*, *NFE2L2*/*KEAP1*/*CUL3*, *PI3K*/*AKT* and *SOX2*/*TP63*/*NOTCH1* pathways and shared many gene mutations in common with head and neck squamous cell carcinomas without evidence of human papilloma virus infection, including *PIK3CA*, *PTEN*, *TP53*, *CDKN2A*, *NOTCH1*, and *HRAS* [98, 99].

In esophageal adenocarcinoma (EAC), significant mutations of *TP53* (71%) and *CDKN2A* (14%) have been reported [100], and these results were consistent with the prominence of TP53 and CDKN2A mutations in Barrett’s esophagus, a precancerous condition of EAC. Likewise, the prominence of TP53 mutation was observed (91%) in esophageal squamous cell carcinoma (ESCC). In the latest detailed molecular classification, ESCC was classified into a high *TP53* mutation group, which possess similar somatic alterations to lung SqCC and head-and-neck SCC, and a low *TP53* mutation group, which has a higher mutation rate of *PIK3CA* and *SMARCA4* [100].

Nearly half (49%) of urothelial bladder cancers had *TP53* mutations, and 76% of the cancers had inactive TP53 functions [101] because their relationship with the amplification (9%) and overexpression (29%) of *MDM2* was mutually exclusive.

### Treatment strategy for *TP53* mutant cancer

**Figure 5:**
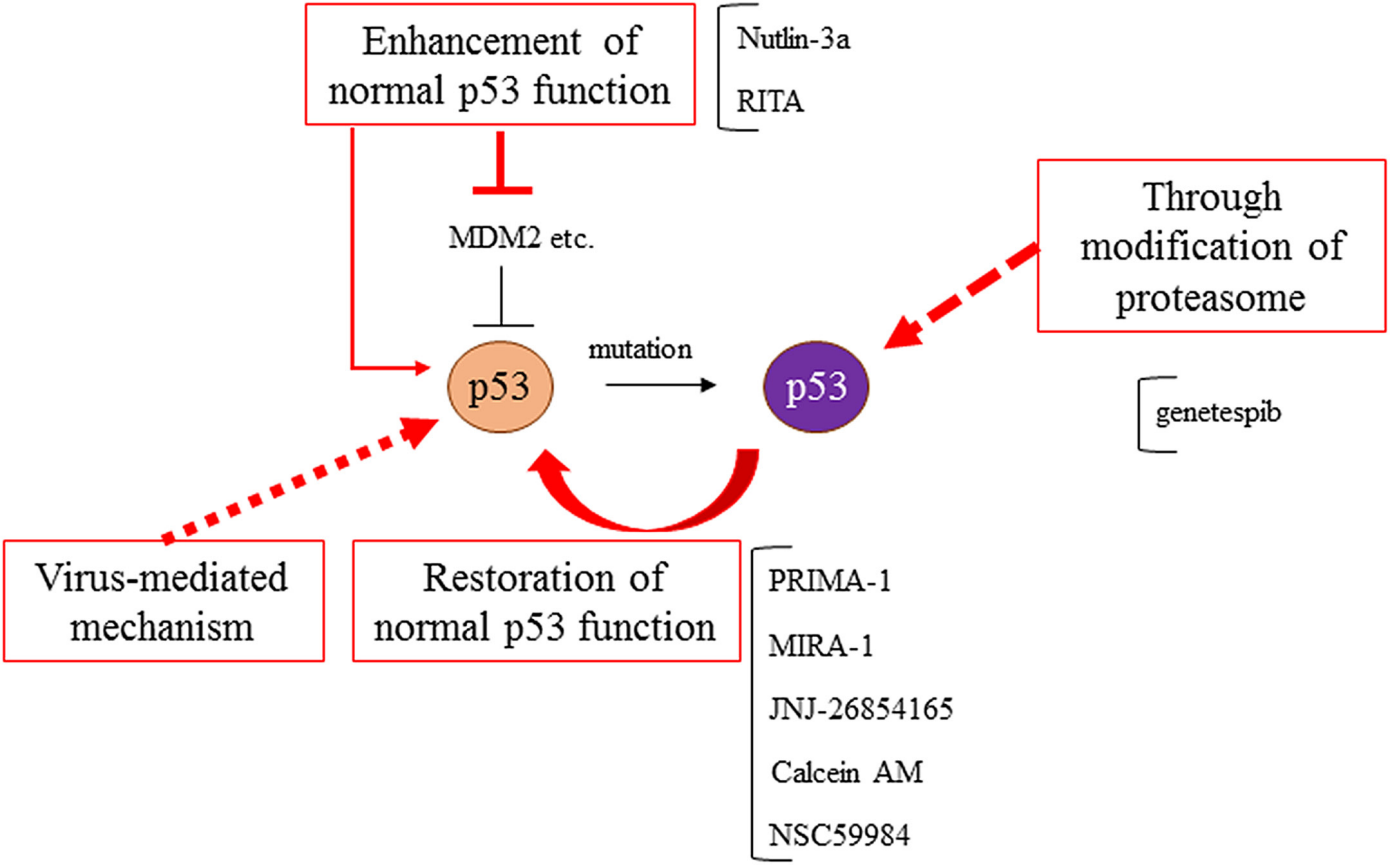
Mutant p53s targeted therapies Treatment strategies for mutant p53s have been developed, such as the restoration of normal p53 function, through the modification of proteasome and autophagy, the enhancement of normal p53 function, and virus-mediated mechanisms.

Mutant *TP53* could therefore be a therapeutic target. Molecular target drugs often exert their effects by suppressing oncogenes that are overexpressed in cancer cells, and oncogenes tend to be the therapeutic targets. In terms of *TP53*-targeted therapy, however, both WT and mutant *TP53* gene-targeted treatments have been proposed [102]. When grouping these treatments according to therapeutic strategy, they can be classified according to 4 objectives: 1) restoration of the normal p53 function lost by genomic mutation [103]; 2) direct attack on p53-deficient cells [104]; 3) enhancement of normal p53 function [105]; and 4) mimicking DNA damage with a virus [106] (Table 1). Many studies examining these possibilities and problems are ongoing, and some interesting studies have successfully achieved their objectives. The major mutant p53 target strategies are shown in Figure 5.

**Table 1:** Treatment strategies targeting mutant p53 cells

Therapeutic concept	Target	Reference No.
Restration of normal p53 function	mutant p53 cell	103, 108, 122, 125
Direct attack to mutant p53 cell	mutant p53 cell	104
Enhancement of normal p53 function	normal p53, MDM2, E6, TopBP1	105, 118, 119, 123, 124
Mimicking of DNA damage using a virus	mutant p53 cell (DNA)	106

PRIMA-1 has been identified as a small molecule that restores transcriptional activity, DNA binding ability, and the structure of WT p53 and that induces mutant p53-dependent apoptosis [107]. PRIMA-1^MET^, or methylated PRIMA-1, contributes to the restoration of WT p53 function through a variety of mechanisms [108]. Currently, there is an ongoing phase II trial examining PRIMA-1, and anti-tumor effects against various cancers have been reported [73, 109–117]. Surprisingly, PRIMA-1^MET^ exerts tumor suppressive effects in various cancers and showed a synergistic effect when combined with existing chemotherapy.

Research on p53 activation in cancer cells with normal p53 has led to the generation of Nutlin-3a and RITA (reactivation of p53 and induction of tumor cell apoptosis) [118, 119]. Nutlin-3a inhibits the binding site of MDM2-p53, promotes p53 function, and increases chemotherapy-induced apoptosis in cancer cells lacking functional p53 by activating E2F2, while RITA avoids MDM2-induced p53 repression by binding to p53 [119]. A phase I trial of Nutlin has been started [120].

Other studies have identified factors that restore normal p53 function [121], such as MIRA-1 [122], JNJ-26854165 [123], Calcein AM [124], and NSC59984 [125]; thus, various clinical trials are expected in the future. MIRA-1 induces apoptosis via the restoration of p53-dependent transcriptional transactivation, such as *p21*, *MDM2*, and *PUMA*, by shifting the equilibrium between the native and unfolded conformation of p53 toward the native conformation [122]. JNJ-26854165 induces p53-mediated apoptosis in leukemia cells and has the potential to be an attractive chemotherapeutic as either a single agent or in combination with AraC or anthracyclines [123]. Calcein AM blocks the oligomerization of TopBP, a key mediator for the oncogenic GOF activity of mutant p53, and blocks p53 binding, resulting in the reactivation of E2F1-dependent apoptosis and a GOF of mutant p53 [124]. Especially, NSC59984 has been reported to exert interesting functions that not only restore normal p53 function through p73 activation, but also deplete the non-canonical functions of mutant p53 via MDM2 and ubiquitin-proteasome activation. On the other hand, the Hsp90 inhibitor ganetespib showed a therapeutic effect only for mutant p53 in the context of an HSP90/HDAC6 chaperone mechanism related to the stability of mutant p53 [126]. Genomic therapy using a recombinant oncolytic adenovirus incorporating WT p53 has been attempted in clinical studies in China [127]. Hence, various studies targeting mutant p53 and its correlation with a poor prognosis have been progressing.

### Future prospects

The functions and mechanisms of the *TP53* gene have gradually become clear over a period of more than 30 years. However, there are still many areas that lack clarity. The next strategy for cancer treatment is expected to become the “Precision Medicine Initiative” announced by the President of the United States, Barack Obama, in 2015. This strategy aims to establish optimal cancer prevention and treatment by grouping patients according to not only genomic phenotypes, but also environments and lifestyles based on information obtained from a cohort study of more than 100 million people. Even using this new approach, the *TP53* gene and its mutations will never be excluded from the interest of cancer researchers, since they have a deep impact on molecular carcinogenesis and the potential for new cancer treatments. We have high expectations that research on the *TP53* gene will continue to advance steadily.

## Abbreviations

GOF: gain of function
WT: wild type
PTMs: post-transcriptional modifications
CIN: chromosomal instability
MSI: microsatellite instability
CIMP: CpG island methylation phenotype
AID: activation-induced cytidine deaminase
CRC: colorectal cancer
GC: gastric cancer
GS: genomic stable
SHM: super high methylation
SCCs: squamous cell carcinomas
ACs: adenocarcinomas
EAC: esophageal adenocarcinoma
ESCC: esophageal squamous cell carcinoma
